# COVID-19, Patents and the Never-Ending Tension between Proprietary Rights and the Protection of Public Health

**DOI:** 10.1017/err.2020.24

**Published:** 2020-04-11

**Authors:** Enrico BONADIO, Andrea BALDINI

**Affiliations:** * Reader in Intellectual Property Law, City, University of London, UK; email: enrico.bonadio.1@city.ac.uk.; ** Director of NJU Center for Sino-Italian Cultural Studies, Nanjing University, China; email: andrea.baldini@nju.edu.cn.

In January 2020, Chinese researchers at the Wuhan Institute of Virology filed for a patent covering the use of remdesivir, an experimental antiviral drug, to treat COVID-19. Normally, this might be cause for celebration: COVID-19, a deadly pneumonia-like disease caused by the novel coronavirus, has so far killed thousands of people worldwide and sickened many more, sending researchers scrambling to develop an effective treatment.^1^ Only, the Wuhan Institute of Virology did not develop remdesivir. The drug was researched and produced by Gilead Sciences, a California-based pharmaceutical company, which had filed patent applications at several patent offices, including in China, covering a “method for treating Arenaviridae and coronaviridae virus infection”.^2^

The remdesivir was originally developed to treat Ebola virus disease (EVD). From November 2018 to August 2019, the drug underwent a Phase 3 experimentation trial.^3^ A relatively large sample of patients with EVD were treated with remdesivir, which appeared less effective than other drugs. However, in early 2020, scientists suddenly became interested in some studies indicating that Gilead’s product could have activity against coronaviruses such as MERS and, hopefully, the rapidly spreading COVID-19.^4^

The Wuhan Institute of Virology’s decision, made in the midst of a rapidly escalating health crisis, to claim rights over an unproven use of the drug was heavily criticised.^5^ It is worth noting that the Wuhan Institute of Virology’s patent application was filed before scientists started experiments investigating the effectiveness of remdesivir against COVID-19. In effect, the Wuhan Institute of Virology’s first *in vitro* studies suggesting that both remdesivir and an antimalarial drug called chloroquine could effectively inhibit COVID-19 was published in early February 2020.^6^ The earliest Phase 3 studies of remdesivir in COVID-19-infected patients started after the publication of that study.^7^

Given the scale of the COVID-19 pandemic, one would expect a virology institute located at its original epicentre to devote its resources and energy to containing the spread of the underlying virus, or perhaps to researching new therapies and vaccines – not to patenting (supposedly new) uses for drugs it neither developed nor tested. Though legally admissible, the Wuhan Institute of Virology’s decision to seek a patent in this case is ethically questionable and may have a negative impact on China’s public health and medical research cooperation efforts.

In response to the public outcry, the Wuhan Institute of Virology defended its patent application by claiming it was made in the national interest. It added that it would be willing to forgo enforcing its patent rights if foreign pharmaceutical companies – in this case, Gilead – collaborate with Chinese authorities to stop the pandemic.^8^ But this argument is weak. If the Wuhan Institute of Virology was really only concerned about public health and access to vital drugs in an emergency, there is already a mechanism that gives countries intellectual property (IP) flexibility in just such an event: compulsory licenses. While controversial, compulsory licenses allow eligible drug-makers to legally manufacture and sell copycat versions of patented drugs during national emergencies, public health crises or in other instances of extreme need. As a form of compensation for the original patent holder, the competent authority – in China’s case, it would be the National Intellectual Property Administration – would require manufacturers to pay a “fair market price” for the drug.^9^

Compulsory licences are explicitly allowed under Articles 48–50 of Chinese patent law, although the country has yet to issue one, and they are in line with the World Trade Organization’s (WTO) Trade-Related Aspects of Intellectual Property Rights (TRIPS) Agreement, which outlines global standards for protecting IP rights. The 2001 Doha Declaration on the TRIPS Agreement and Public Health, adopted by the WTO Ministerial Conference, confirmed that compulsory licences can be used subject to certain conditions. And where they have been granted, they have led to significant reductions in drug prices. In 2012, for example, the Indian generic drug-maker Natco was granted a compulsory licence for sorafenib, an anticancer drug, after that country’s patent office ruled that Bayer AG, sorafenib’s patent holder, had not done enough to make the drug available to Indian citizens. Natco was required to pay 6% royalties to the German company – a figure based on United Nations (UN) guidelines – and proposed selling its version for 97% below Bayer’s price.^10^

Given the scale of the ongoing health crisis, a Chinese company filing for a compulsory licence would not necessarily be perceived as an attempt to unjustifiably circumvent Gilead’s patent rights. After all, in the wake of the coronavirus crisis, in March 2020, Israel issued a compulsory licence in relation to Kaletra, an HIV medicine that is currently being tested for effectiveness in the treatment of COVID-19. The patent is owned by the US pharmaceutical company AbbVie, and the licence will allow Israel to import the generic version of Kaletra produced by the Indian company Hetero.^11^ In addition, the Chilean parliament^12^ and Ecuador’s National Assembly^13^ have adopted resolutions that would pave the way for the issuance of compulsory licences to tackle the coronavirus outbreak, and the German government has started plans to limit patent rights in view of this pandemic.^14^ In North American, Canada is also following this lead.^15^

The unusual strategy that the Wuhan Institute of Virology settled on – seeking to patent an untested use of a drug in such a way that it might interfere with patent rights owned by a foreign corporation – has raised hackles. Some critics have accused the institute of trying to get out of paying Gilead a licencing fee should a compulsory license be granted.^16^ Others have read its move as an attempt to secure bargaining chips in possible upcoming pricing negotiations with the company.^17^

Another Chinese entity has come under fire from patent advocates: the Suzhou-based BrightGene Bio-Medical Technology, which may also be in for a fight after confirming that it had synthesised remdesivir’s active ingredient without first obtaining permission from the patent holder. Though the company says it is interested in setting up a voluntary licensing agreement with Gilead at some point in the future, it also claims that its work has not infringed that company’s patent rights because the final product is not being sold on the market.^18^ But this is highly debatable. Indeed, under both international and national patent laws, manufacturing a patented medicine, even if it is not yet on sale, still amounts to patent infringement.^19^

There are real risks to the strategies adopted by the Wuhan Institute of Virology and BrightGene. In particular, they may raise international companies’ suspicions regarding their Chinese peers. This in turn may hamper vital research cooperation between China and the world. Gilead, for example, has offered samples of remdesivir for use in clinical trials during the current outbreak. Other leading international pharmaceutical firms currently working on vaccines for COVID-19, such as Johnson & Johnson and GlaxoSmithKline, may have less incentive to do so in the future if they believe China will not support their IP claims. A collaborative attitude has also been shown by AbbVie, which, in March 2020, informed that due to the current health emergency it would stop enforcing its Kaletra patent anywhere in the world, as well as in relation to the treatment of HIV.^20^

The Wuhan Institute of Virology and BrightGene are not the only entities that have been criticised. Gilead itself came under fire after its version of remdesivir obtained in March 2020 the orphan drug designation from the US Food and Drug Administration. Under the US Orphan Drugs Act, such a designation gives a seven-year market exclusivity period, as well as tax and other incentives for pharmaceutical companies that produce medicines for rare diseases that impact fewer than 200,000 people. Gilead was reprimanded for applying for such status and thus seeking exclusive rights “despite calls for solidarity” to face the pandemic.^21^ After criticism, Gilead informed the public that it had requested to rescind the orphan drug designation.^22^

As previously mentioned, one may argue that these behaviours are ethically questionable – and in particular that the patent system should not be used to make access to drugs more difficult, especially during a pandemic. Can we really justify IP laws that are used in a way that limits the availability of medicines and aims at increasing profits in times of health emergency? This moment of crisis is teaching us a clear lesson in matters of the philosophical justifications of IP: egoistic theories are incapable of offering convincing arguments grounding IP protection.^23^ In effect, theories that consider personal gain (both in terms of existential self-realisation^24^ or economic gain^25^) as the only legitimate source of an ethical defence of IP and as an overarching reason in cases of conflicts between individual and societal well-being appear untenable. The COVID-19 pandemic shows the essential interconnectedness of human beings as a community of unity, where individual happiness becomes possible only in cases where a certain level of welfare is collectively shared.

In this sense, plausible justifications of IP protection must address the relationship between individual and collective needs and concerns. Utilitarianism does just that: it offers an argument in favour of IP that recognises egoistic motives, though within a larger altruistic framework where societal utility functions as the ultimate goal of our practices. Traditional versions of this argument suggest that incentives to authors and inventors are instrumental in maximising social utility, which is the key principle of utilitarian ethical theories.^26^ In this sense, IP protection rewards innovators, who are then stimulated to invest more time in inventive and creative activities. This in turn favours optimal social utility. Rewards are only justified in terms of the good that, indirectly, they bring to the whole of society. In this view, therefore, personal gain is simply a means to a higher purpose. Clearly, such a utilitarian approach fits perfectly into the pharmaceutical industry’s incentive-focused narrative of “if patents for drugs are not available, research-and-development efforts will be discouraged”.

Traditional forms of utilitarianism show limitations insofar as they tend to identify innovators as individual subjects (either persons, institutions or companies). However, as efforts from the scientific community during this time of crisis clearly show, the process of discovery is often distributed and may very well profit from collaborations between different entities. In this sense, some innovators may very well be collective rather than individual subjects, and their activity should be protected. Therefore, among the less obvious implications of altruistic justifications of IP we have the following: IP should establish favourable conditions for collaboration and exchange and for speeding up the process of scientific discovery.

It is often difficult to balance these two opposing forces. The AIDS and HIV crisis of the 1980s and 1990s already showed us how patent laws may be used to oppose policies adopted by democratically elected governments to balance patent rights and make drugs more affordable. Indeed, in 1998, a group of pharmaceutical companies took the South African government to court to try to stop it introducing legislation aimed at reducing the price of medicines, the main objection being that the 1998 South African Medicines Act had arbitrarily reduced patent protection (the legal action was abandoned in 2001).^27^

What these (old and recent) disputable behaviours teach us is that IP protection and in particular patent regimes must be managed with great care, as well as a willingness to occasionally set aside financial considerations in favour of ethical or moral concerns, especially when it comes to facing unprecedented global health emergencies such as the COVID-19 pandemic. While IP laws are certainly crucial as they incentivise the development of (often) vital drugs, they are far from perfect, and may very well require further adjustment or reform to meet overarching public interests. The solution is not to erode the mutual trust required to make international public health cooperation work.

## Footnotes

1 Y Roulin, “Wuhan’s Much-Maligned Virology Institute Seeks Patent on US Drug,” *Sixth Tone*, 11 February 2020 <http://www.sixthtone.com/news/1005169/wuhans-much-maligned-virology-institute-seeks-patent-on-us-drug>.

2 See Chinese patent application No CN108348526A, available at <https://patents.google.com/patent/CN108348526A/en?oq=US2017071964>.

3 S Mulangu et al, “A Randomized, Controlled Trial of Ebola Virus Disease Therapeutics” (2019) 381 New England Journal of Medicine 2293 3177495010.1056/NEJMoa1910993PMC10680050.

4 TP Sheahan et al, “Comparative Therapeutic Efficacy of Remdesivir and Combination Lopinavir, Ritonavir, and Interferon Beta against MERS-CoV” (2020) 11 Nature Communications 222 10.1038/s41467-019-13940-6PMC695430231924756.

5 W Haiyun, “China to Begin Testing Ebola Drug on Coronavirus Patients,” *Sixth Tone*, 3 February 2020 <http://www.sixthtone.com/news/1005155/china-to-begin-testing-ebola-drug-on-coronavirus-patients>.

6 M Wang et al, “Remdesivir and Chloroquine Effectively Inhibit the Recently Emerged Novel Coronavirus (2019-NCoV) *In Vitro*” (2020) 30 Cell Research 2693202002910.1038/s41422-020-0282-0PMC7054408.

7 <https://clinicaltrials.gov/ct2/show/NCT04252664?term=remdesivir&draw=2&rank=2>; <https://clinicaltrials.gov/ct2/show/NCT04257656?term=remdesivir&draw=2&rank=1>.

8 A Wininger, “Wuhan Institute of Virology Applies for a Patent on Gilead’s Remdesivir,” *The National Law Review*, 6 February 2020 <https://www.natlawreview.com/article/wuhan-institute-virology-applies-patent-gilead-s-remdesivir>.

9 See the webpage <http://english.cnipa.gov.cn>.

10 E Bonadio, “Compulsory Licensing of Patents: The Bayer/Natco Case” (2012) 10 European Intellectual Property Review 719 .

11 S Kass, “Israel Defies AbbVie IP to Import Generic Drugs for COVID-19”, *Law360*, 19 March 2020 <https://www.law360.com/articles/1255079/israel-defies-abbvie-ip-to-import-generic-drugs-for-covid-19>.

12 See Resolution 896 adopted by the Chile Chamber of Deputies on 17 March 2020, available at <https://www.keionline.org/chilean-covid-resolution>.

13 See the webpage <https://www.keionline.org/32429>.

14 T Musmann, “German Government Plans Possibilities to Limit Patents in View of Corona Pandemic”, *Kluwer Patent Blog*, 24 March 2020 <http://patentblog.kluweriplaw.com/2020/03/24/german-government-plans-possibilities-to-limit-patents-in-view-of-corona-pandemic>.

15 R O’Neill, “Canada Authorises Compulsory Licences for COVID-19”, *Life Sciences Intellectual Property Review*, 27 March 2020 <https://www.lifesciencesipreview.com/news/canada-authorises-compulsory-licences-for-covid-19-shortfalls-3972>.

16 J McDonald and LA Johnson, “Chinese Scientists Ask for Patent on US Drug to Fight Virus”, *Associated Press News*, 6 February 2020 <https://apnews.com/1fe943717b56b56cce5e733790f016dd>.

17 S Decker, D Lyu and H Fan, “Gilead Declines on HIV Patent Fight Loss, China IP Interest”, *Bloomberg*, 5 February 2020 <https://www.bloomberg.com/news/articles/2020-02-05/gilead-declines-after-hiv-patent-fight-loss-china-ip-interest>.

18 A Liu, “Chinese Firm Copies Gilead’s Remdesivir, the Most Promising Drug against the New Coronavirus”, *FiercePharma*, 12 February 2020 <https://www.fiercepharma.com/pharma-asia/chinese-firm-copies-gilead-s-remdesivir-most-promising-drug-against-new-coronavirus>.

19 See, for example, Art 28 of the WTO TRIPS Agreement.

20 E ‘t Hoen, “Covid-19 and the Comeback of Compulsory Licensing”, *Medicines Law and Policy*, 23 March 2020 <https://medicineslawandpolicy.org/2020/03/covid-19-and-the-come-back-of-compulsory-licensing>; DP Mancini, “AbbVie Drops Patent Rights for Kaletra Antiviral Treatment”, *Financial Times*, 23 March 2020 <https://www.ft.com/content/5a7a9658-6d1f-11ea-89df-41bea055720b>.

21 DP Mancini, “Gilead Criticised Over ‘Orphan Status’ for Potential Virus Treatment”, *Financial Times*, 24 March 2020 <https://www.ft.com/content/9fea4f1c-6dba-11ea-89df-41bea055720b>.

22 See Gilead’s press release at <https://www.gilead.com/-/media/gilead-corporate/files/pdfs/company-statements/remdesivir-orphan-drug-designation.pdf?la=en&hash=ED14BC7B26E2FEAA2E31E7741A8C9692>.

23 For an instructive survey of the philosophical justifications of IP, see A Moore and K Himma, “Intellectual Property” in EN Zalta (ed.), The Stanford Encyclopedia of Philosophy (Stanford, CA, Metaphysics Research Lab, Stanford University 2018 ) <https://plato.stanford.edu/archives/win2018/entries/intellectual-property>.

24 GWF Hegel, Elements of the Philosophy of Right, A Wood (ed.) (Cambridge, Cambridge University Press 1991 ).

25 J Locke, The Second Treatise of Government, P Leslett (ed.) (Cambridge, Cambridge University Press 1988 ).

26 TG Palmer, “Are Patents and Copyrights Morally Justified – The Philosophy of Property Rights and Ideal Objects Symposium on Law and Philosophy” (1990) 13 Harvard Journal of Law & Public Policy 817 .

27 WW Fisher III and CP Rigamonti, “The South Africa AIDS Controversy: A Case Study in Patent Law and Policy”, *Cyber Harvard*, 10 February 2005 <https://cyber.harvard.edu/people/tfisher/South%20Africa.pdf>.

